# Recruiting participants for an international mHealth study via Facebook Ads: Experiences from the Untire App RCT

**DOI:** 10.1016/j.invent.2021.100362

**Published:** 2021-01-07

**Authors:** Simon S. Spahrkäs, Anne Looijmans, Robbert Sanderman, Mariët Hagedoorn

**Affiliations:** a Department of Health Psychology, University of Groningen, University Medical Center Groningen, the Netherlands; b Department of Psychology, Health & Technology, University of Twente, the Netherlands

**Keywords:** Online recruitment, Facebook Ads, Reach, Costs, mHealth, Psycho-oncology

## Abstract

**Introduction:**

Social media recruitment via Facebook Ads seems to be a promising method for large-scale international trials examining the effectiveness of mHealth interventions. However, little is known about this method in terms of strategy, reach, and costs in the context of psycho-oncology. This paper presents the results of the recruitment strategy that was applied in the Untire app study and shows how many participants could be reached using advertisements (i.e., Ads) on Facebook, who participated, and what it cost.

**Method:**

The Untire app study is a randomized controlled trial targeted at cancer patients and survivors across four English-speaking countries (i.e., Australia, Canada, the U.K., and the U.S.A.). Reach was assessed by the number of people who were shown the Ads, who clicked on the Ads, and completed study assessments. Demographic characteristics were gathered from Facebook Ads Manager and from online study assessments to describe who was reached. Costs were assessed by the budget spent and the cost per click for Ads, for reaching the study's landing page, and for completing study assessments. To conduct a powered RCT, we needed 164 12-weeks assessments in both the intervention and the control group.

**Results:**

From March till October 2018, we used 76 Ads, which were presented to 1.2 million people. 37.376 persons clicked on the study link in the Ads, resulting in 755 baseline completers. Most participants were female (92%), middle-aged (55.5 ± 9.79), and came from the U.K. (72%). The total Facebook advertisement costs from March till October 2018 were €17 k, resulting in an average cost of €0.45 per click on the Ads, €5.55 on average for a person reaching the study's landing page, and €14.89 on average per eligible participant. The costs for every baseline and 12-weeks completer were €22.42 and €47.69, respectively.

**Discussion:**

Reaching participants for international mHealth studies in psycho-oncology via Facebook Ads has potential but is costly. The key to reducing costs lies in constant optimization and testing of Ads and refinement of target audience characteristics.

## Highlights

• Reaching (ex) cancer patients via Facebook Ads for mHealth studies has potential.
• Online recruitment is useful in reaching participants who represent end-users.
• Costs of reaching (ex) cancer patients below 25 years were on average twice as high.
• Reaching participants via Facebook Ads might be resource-intensive
• Costs of online recruitment can be reduced via optimization and testing of Ads.

## 1 Introduction

Nowadays, patients are increasingly seeking support and practical advice on their health online, via their smartphones (Kuehn, 2011; Riggare, 2018; World Health Organization, 2011). Stand-alone interventions offered via mobile health (mHealth) apps can provide scalable healthcare for many patients worldwide (Seiler et al., 2017). With more than 350.000 mHealth apps being available in the worldwide app stores (Byambasuren et al., 2019), there is a clear need for clinical validation of the effectiveness of such mHealth apps in clinical trials (Chib and Lin, 2018), and feasible recruitment strategies for reaching participants for such trials (Watson et al., 2018). Since mHealth trials deliver the intervention online via a smartphone, it seems plausible to also recruit participants online, for example, via social media online advertisements (Ads).

Ads disseminated via social media like Facebook might provide an effective solution to reach out to a large number of potential mHealth users (Murray et al., 2009) and research participants in clinical trials validating these mHealth interventions (Dizon et al., 2019). Recruitment via Facebook could entail various advantages, including anonymity, confidentiality, convenience, high potential reach, cost-effectiveness, usability, and the capacity for targeting hard-to-reach populations, including isolated and minority populations, as well as populations with sensitive and stigmatizing mental health issues (Batterham, 2014; Frandsen et al., 2016). Facebook is currently the most popular social media platform in the Western world, with 2.07 billion monthly users worldwide (Wozney et al., 2019). Despite several advantages, recruiting participants into clinical trials online can be challenging, entailing a higher risk of selection bias, study delay, and study termination as there is no personal contact (Darmawan et al., 2020; van Gelder et al., 2019). Before deciding to use Facebook as a recruitment strategy for a particular study, more information on experiences of similar studies would be helpful.

A review by Topolovec-Vranic and Natarajan (2016) comparing social media recruitment (e.g., via Facebook, Instagram, Twitter) to traditional recruitment methods (e.g., community-based organizations, print-media, friends referral, word of mouth) identified mixed results: in 12 studies, social media recruitment was the somewhat more effective recruitment method in terms of the number of participants recruited within a specific time period, whereas in 15 studies, traditional methods were more effective. The review included eight online interventions, of which only one was assessing the effectiveness of a mHealth intervention (Partridge et al., 2015). In addition, a review by Thornton et al. (2016) included 110 studies that used Facebook for recruiting participants in health, medical or psychosocial research, of which 88 studies were cross-sectional survey studies, 17 were trials, and 7 were longitudinal surveys. Of the studies that examined the sample's representativeness (*n* = 16), 86% concluded that their Facebook-recruited samples were similarly representative as samples recruited via traditional methods. In sum, the effectiveness and representativeness of recruitment via traditional methods and Facebook seem similar.

A potential advantage of online recruitment is cost-effectiveness. To obtain the required sample size for an empirical study, social media advertisement campaigns are regularly large-scale, and therefore, financial costs are often difficult to estimate. Costs per randomized participant have been shown to differ enormously, e.g., 1.51 USD (Partridge et al., 2015), 2.01 USD (Lee et al., 2020), or 172.76 USD (Heffner et al., 2013). The review by Thornton et al. (2016) calculated that costs for the recruitment of research participants ranged between 1.36 USD and 110 USD per completed baseline questionnaire (Mean = 17.48 USD, S.D. = 23.06 USD), suggesting overall cost-effectiveness, despite the enormous variability in costs.

In sum, the literature suggests that online recruitment via Facebook can be a cost-effective and feasible method of recruiting a sufficient number of study participants, particularly for hard-to-reach populations from all over the world and for specific or rare health conditions (Thornton et al., 2016). The literature also emphasizes that studies should report on their recruitment strategy and costs when using Facebook (Thornton et al., 2016). In our large scale international randomized controlled trial (RCT), we evaluated the effectiveness of the Untire self-management app. The Untire app was developed to improve fatigue and quality of life of cancer patients and survivors (i.e., people who have received a diagnosis and treatment for cancer in the past) who felt moderately to severely fatigued (Spahrkäs et al., 2020a, Spahrkäs et al., 2020b). Since cancer survivors might be challenging to reach for study participation after they have completed their treatment (Hulbert-Williams et al., 2019; van Lankveld et al., 2018), we decided to recruit study participants using Facebook Ads. In addition, since the intervention (i.e., app) is delivered via the smartphone, Facebook Ads recruitment seemed like a promising strategy. Since only a small number of studies in the literature have recruited cancer patients and survivors via Facebook Ads, or recruited participants for mHealth trials, we will here describe our recruitment strategy. By sharing our experiences, we hope to guide other researchers who plan to recruit cancer patients and survivors through Facebook Ads Manager for mHealth research.

### 1.1 Objectives

#### 1.1.1 How many participants can be reached and who participates?

The first objective is to describe the recruitment process in terms of strategy, the number of people reached, and the study participants' composition. Comparing demographic characteristics of participants reached via Facebook campaigns with characteristics of study participants (i.e., age, gender, country) might provide valuable insights into differences in Facebook targeting and eventual study participation, as well as insights into potential end-users of such mHealth interventions.

#### 1.1.2 What are the costs of recruitment?

The second objective is to evaluate the recruitment costs, given the number of people reached by Facebook Ads and participating in the study.

## 2 Method

### 2.1 Study design and setting

All data are derived from the Untire app study, which is an international large-scale waiting-list RCT. Details are provided in the protocol (Spahrkäs et al., 2020a) and a publication regarding the app's effectiveness (Spahrkäs et al., 2020b). In the Untire app study, potential participants received Facebook Ads to join a study on ‘Beating Cancer Fatigue’ on their personal Facebook News Feed when they previously had shown interest in topics related to cancer, survivorship, and patient support. With the Facebook Ads Manager platform, we collected data on recruitment. Data for examining the app's effectiveness were collected using online assessments (Questback's EFS-survey software).

The Untire app study was approved by the Medical Ethical Committee (METc) of the University Medical Center Groningen (UMCG), the Netherlands, and we also received either ethical approval or a waiver from authorized institutions in the four English speaking countries targeted (i.e., Australia, Canada, United Kingdom, and the United States).

### 2.2 Study population

The target population of the RCT included both cancer patients and survivors of ≥18 years, who experienced persistent fatigue at moderate or severe levels (i.e., an average composite score of ≥3 on items 1–3 of the Fatigue Symptom Inventory (Hann et al., 1998, Hann et al., 2000) and owned a smartphone, tablet or iPad (i.e., iOS/Android). Exclusion criteria were a diagnosis of and receiving treatment for a severe mental disorder (i.e., major depression, psychotic disorder, anxiety disorder, or addiction) as well as having a diagnosis of myalgic encephalomyelitis/chronic fatigue syndrome (ME/CFS) or fibromyalgia (F.M.). All inclusion and exclusion criteria were self-reported. Based on our study target population, we chose the Facebook recruitment strategy described below. We used Facebook to recruit potential participants, but the eligibility of participants was checked at the study's landing page.

### 2.3 Procedure and recruitment strategy

**Fig. 1:**
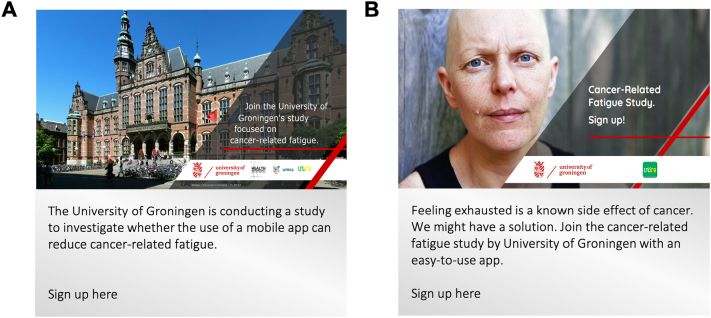
(A) Facebook Ad example 1; (B) Facebook Ad example 2.

In February 2018, our research team launched a new Facebook page. Via this Facebook page, we executed Facebook's Ads Manager. Facebook Ads manager comprises (1) campaigns, (2) Ad-sets, and (3) Ads. Every campaign had a single objective (i.e., generating reach, likes, etc.) and consisted of one or more Ad-sets (i.e., groups of Ads that share settings for how, when, and where to run). At Ad-sets, the target audience (based on Facebook location, gender, age), placements (e.g., placing Facebook Ads on Instagram), and a budget and schedule could be defined. Every Ad-set consisted of one or more Ads (i.e., images with text; see Fig. 1 for two examples of our Ad creatives, further see Supplement 1 for additional samples of Ad creatives used over time with varying pictures, texts, and formats). The text in each Ad described the study briefly and provided a hyperlink to the study's landing page (i.e., first page) in Questback survey software.

Over eight months (March – October 2018), we created a total of 26 campaigns and explored which ones brought the best results with our target audience, at which time, and costs. Specifically, we used more than 66 Ad-sets to test different demographics determining over time who is most likely to respond by clicking the link. We used 76 Ads and explored which of the 18 texts and 16 images we had created worked best. The Facebook Ads Manager platform provides researchers with the ability to target variables like age, location, interests, and behaviors of those shown the Ads (Tsai et al., 2019). Facebook Ads were first disseminated to target audiences with similar interests to those who are a member of Facebook groups/pages related to cancer and survivorship (e.g., ‘Cancer Awareness,’ or ‘MacMillan Cancer Support’). Subsequently, we targeted more broadly people with a so-called interest in ‘cancer’ or ‘breast cancer,’ leading to larger audiences and fewer costs. In the beginning, we used advertisement campaigns with unrestricted audiences. Based on participant characteristics of those who showed interest (i.e., clicked on the Ads), comparable participants (i.e., lookalike audiences) were approached in subsequent advertisement campaigns and Ad-sets. The Ad-sets were continuously adapted to reach participants with the highest probability of link-clicks.

Every time an Ad was clicked, our Facebook account was charged, reducing our credit. We switched Ads on/off frequently throughout the recruitment period based on their effectiveness, as shown by the average cost per click (CPC). Once the CPC rate of Ads, Ad-sets, or campaigns ascended steeply (i.e., the advertisement's costs did outweigh the number of clicks), we switched these off. Our Facebook recruitment budget was about €18.000. We aimed for the lowest CPC to have the best value for money, although we had not specified a pre-determined CPC limit.

Facebook users who clicked on our Ads were taken to our study's landing page. This page contained a brief description and video of the app, a link to frequently asked questions (FAQ), and access to the eligibility screening. Interested respondents could also contact the research team. Once eligible, participants received an information letter and gave digitally informed consent. Hereafter, participants completed a baseline questionnaire (assessing participant characteristics, and among others, baseline levels of fatigue and quality of life), after which they were randomized 2:1 by Questback survey software into the intervention or control group. Participants received invitations to fill out further online study assessments at 4, 8, 12, and 24 weeks after baseline. Sample size calculations showed that we needed 164 completed assessments in the intervention, and 164 completed assessments in the control group to examine the primary outcome at 12-weeks. More information about the study assessments and the randomization procedure can be found in our protocol paper (Spahrkäs et al., 2020a).

### 2.4 Measurements and analyses

#### 2.4.1 Reach

Reach refers to the number of people who received the Ad on their Facebook News Feed (Arigo et al., 2018). The number of participants reached, as well as demographic characteristics, can be found in the Facebook Ads Manager. Further, we explored and compared characteristics (i.e., gender, age, country of residence, and education) of persons reached by Facebook Ads and our study participants' characteristics at the baseline assessment.

#### 2.4.2 Clicks

Once a Facebook user clicked our Ad, the Facebook Ads Manager registered one Facebook Click. Every person who landed on the study's landing page (i.e., first page) and clicked ‘further’ to go to the second page was counted as one Landing Page Click (i.e., they started the survey). We decided to distinguish between the Facebook Click and the Landing Page Click since a vast amount of people who clicked on the Facebook Ad arriving at the study's landing page did not click further to go to the second page but dropped out instead. We collected metrics on the click-through rate (CTR: percentage of Facebook Clicks per persons receiving the Ads on their Facebook News Feed), conversion rate (percentage of surveys started per Facebook Click) as well as completion rate (percentage of surveys completed per Facebook Click) (Lee et al., 2020).

#### 2.4.3 Costs

We calculated the total costs of recruitment spent on campaigns, as well as costs per person per Facebook Click and Landing Page Click. Further, we calculated the costs per person eligible and had signed the informed consent and the costs per person who completed the baseline and 12-week study assessment. It is essential to realize that online recruitment costs using Facebook Ads Manager solely represent payments towards Facebook and do not consider all the working hours of campaign management.

## 3 Results

### 3.1 How many participants were reached, and who participated?

**Fig. 2:**
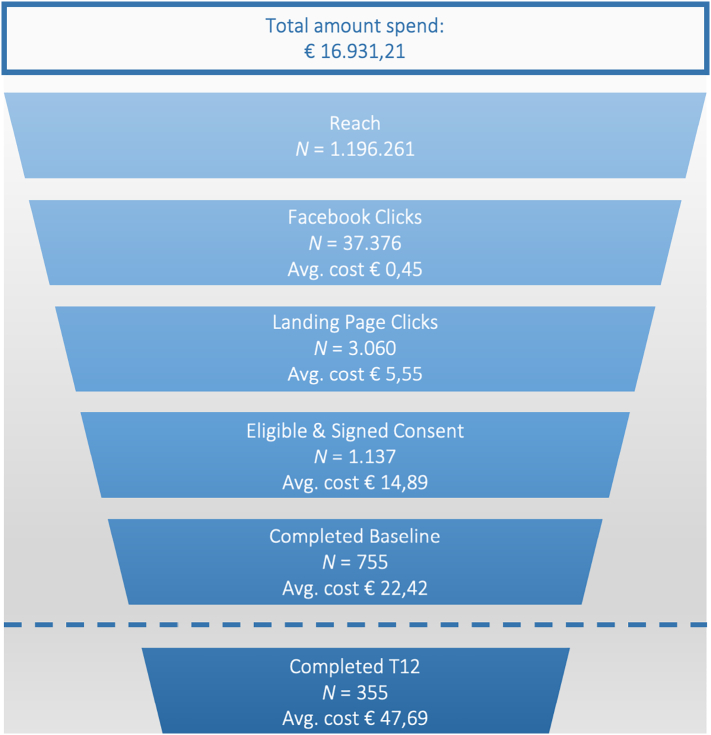
Flow chart of (potential) participants reached and recruitment costs. Total amount spent, average cost per click (Facebook) on advertisement campaign, average cost per click on landing page, average cost per eligible participant, average cost per completed baseline, and completed 12-week assessment.

In a period of 8 months (March – October 2018), our Ads were disseminated about 1.2 million times, resulting in 37.376 Facebook Clicks (CTR = 3%), 3.060 Landing Page Clicks (i.e., survey starts), 755 baseline study assessment completers, and 355 12-week study assessment completers (Fig. 2). At the primary endpoint at 12-weeks, the targeted number of assessments in the control group was reached (*n* = 176), whereas the number of assessments in the intervention group was near the calculated minimum (159 assessments instead of 164). Table 2 portrays the performance of the advertisements according to Facebook and study metrics.

Thirty-five percent of the people who completed the eligibility screening were not eligible and thus could not participate. Eighty percent of the non-eligible participants (*n* = 467) had suffered comorbidities (*n* = 374), which cannot be detected ‘a priori’ by Facebook Ads-sets. Only 1% (*n* = 6) indicated to be <18 years, which is, of course, easy to target with Facebook Ads. Our recruitment strategy was successful in reaching both cancer patients and survivors (*n* = 485 survivors, which is 60% of the baseline participants) while keeping the number of people (*n* = 41) who never have had cancer low (9% of the non-eligible).

During the recruitment period, we found that our Ad-sets aimed at women of 45 years and older led to the highest number of Facebook Clicks compared to men and other age groups (Table 1). We were using this information to tailor the recruitment strategy to approach similar participants (lookalike audiences) in subsequent campaigns, resulting in the fact that the vast majority of study participants were female (96%) and middle-aged (mean age: 56.5 ± 9.76). Further, the campaigns resulted in half of the Facebook Clicks coming from the U.K. (46%), followed by the United States, Canada, and Australia, whereas in the study, participants from the U.K. were overrepresented (72%).

**Table 1:** Demographics of Facebook users who clicked on Ads and demographics of study participants in the Untire study who completed the baseline assessment.

Demographics	Facebook	Baseline assessment
Gender (%)		
Female	93.7	91.4
Male	5.3	8.1
Unknown	1.0	0.5

Age group (%)		
18–24	0.4	0.1
25–34	0.5	1.8
35–44	7.7	7.3
45–54	26.1	43.7
55–64	39.0	35.1
65+	26.6	12.1

Country (%)		
Australia	6.2	6.8
Canada	12.8	6.8
The United Kingdom	46.1	72.0
The United States of America	34.9	13.3
Other	0.0	1.4

Education, N (%)		
Low		18.9
Moderate		38.3
High		42.8

*Note*: Education: Low = Primary school and High school; Moderate = Associative degree or apprenticeship; High = University (Bachelor, Master, or higher).

**Table 2:** Facebook recruitment advertisement performance.

Facebook and study metrics	*N*	%
Reach	1.196.261	
Facebook clicks	37.376	
Click-through rate (CTR)		3.1
Landing page clicks (survey started)	3.060	
Eligible and signed consent	1.137	
T0 survey completed	755	
T0 conversion rate		8.2
T0 completion rate		2.0
T12 completed	355	
T12 required *N*	328	

*Note:* Landing page clicks = survey started; Click through rate = percentage of Facebook clicks per persons receiving the Ads on their Facebook News Feed); conversion rate = percentage of surveys started per Facebook clicks; completion rate = percentage of surveys completed per link clicks.

### 3.2 How much did these Facebook campaigns cost?

**Fig. 3:**
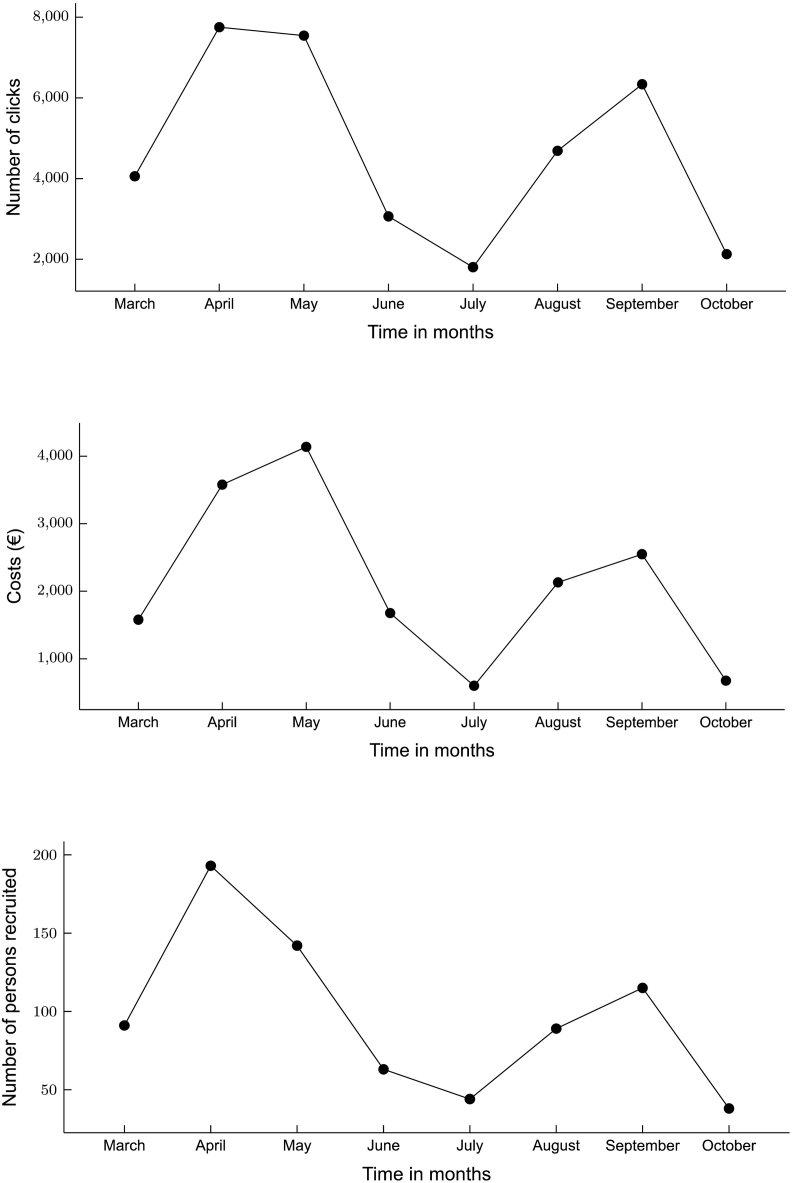
Number of Facebook Ad clicks (March–October 2018), cost of advertisements per month in Euro (€), and number of persons recruited in the study.

The total Facebook campaign costs were €16,931.21 (see Fig. 2).The average costs per eligible participant were €14.89, for every baseline study assessment completer €22.42, and every 12-week study assessment completer €47.69. Fig. 3 presents the fluctuations in costs over the eight-month recruitment period in relation to the recruited participants. Months in which more budget was spent on campaigns yielded a higher number of recruited participants. These costs reflect the actual Facebook costs for advertisements but do not include the costs of working hours for creating and managing advertisement campaigns and responding to comments and questions on social media. We estimate a total of 154 working hours, based on determining strategy (10h), copywriting (4 h), creating visuals (8 h), setting up campaigns (8 h), creating a landing page (12), monitoring and managing comments and questions (104 h: 4 h/week for 26 weeks), as well as evaluation of the strategy (8 h).

## 4 Discussion

### 4.1 Principal findings

This paper aimed to outline our experiences using online recruitment via Facebook Ads in an international mHealth study among cancer patients and survivors by providing insights into the reach and costs of this method. Our data showed that we indeed could reach out to huge numbers (i.e., 1.2 million people received our Ad in their News Feed), albeit the total advertisement cost was high (i.e., around €17 k). Hence, recruitment via Facebook campaigns is not necessarily cheap; in our case, about €14,89 per eligible participant and almost €50 per participant who completed the primary study assessments at 12 weeks. As aforementioned, these costs reflect the actual Facebook advertisement costs but do not include the costs of substantial working hours (i.e., 154 h estimated over 26 weeks) for creating and managing the advertisement campaigns. Furthermore, the creative design of Ads also requires specific skills. On the other hand, costs for creating and managing recruitment materials are not exclusive for online recruitment but are also needed in other (traditional) recruitment methods (Frandsen et al., 2016).

At the start, we distributed the budget for advertisements equally to target younger and older people, men and women, and people living in different English speaking countries. Hereafter, we continuously adapted the advertisement strategy (Ad-sets) to reach participants with the highest probability of Facebook clicks (lookalike audiences). For instance, after the first month of recruitment, we restricted the age range from 18 to 65 to 35–65, since only 9% of the people who clicked were 18–35, and Ad-sets targeting this younger group were exceptionally costly. Specifically, reaching younger baseline participants <25 years costed us on average more than twice as much as reaching baseline participants ≥25 years (average costs per baseline participant EUR 44.67 vs. EUR 22.91, respectively). Similarly, women appeared to be more responsive to our Ads than men and, therefore, were targeted more specifically. We chose to select lookalike audiences based on the lowest CPC continuously. The average CPC in our study was 0.45 EUR (=0.52 USD), which was lower than the CPC among English (0.67 USD), Korean (0.97 USD), and Chinese (0.66–0.79 USD), but not Spanish speaking (0.31 USD) cancer survivors recruited via Facebook in a recent observational web-based survey study (Tsai et al., 2019). However, it is essential to realize that CPC rates can only predict to a limited extent how many participants will start and complete the study after clicking the Facebook Ad, making sample size estimations rather difficult.

Following our recruitment strategy involving the lowest CPC, we recruited a sample representing the group of people most likely to be interested in the mHealth study and are likely to be the future app users (i.e., female cancer patients and survivors aged 35–65). Previous research showing that men generally do not seek as much psychological help as women (Liddon et al., 2018) further supports our inference that especially women between 35 and 65 might be future app users. One of the reasons for an over-representation of females in several health studies with Facebook recruitment might be that a higher percentage of women use Facebook (Whitaker et al., 2017). Further, fewer men may respond to healthcare recruitment in general, which affects both traditional as well as social media recruitment methods (Whitaker et al., 2017). To tackle this issue, men should be motivated and targeted via their social environment, and custom Ads should be used. Tailoring Ad creatives for targeting men vs. women would help to increase reach further since different messages will get noticed by different genders. The finding that participants in our study experience high baseline levels of fatigue and little support to deal with their fatigue further corroborates the notion that our sample represents our target group. In other cases where equal distribution of sample characteristics is desirable, one will need to invest more money in hard to reach groups (e.g., men).

The recruitment strategy to select Ad-sets with lower CPC could automatically result in differences between various countries. While our Ads have reached a similar number of persons from the U.K. and the U.S., many more persons from the U.K. participated in the study. Facebook data suggests that the advertisement CPC rate for Facebook Ads varies between countries (Nanigans, 2016), with a higher average CPC for Australia, followed by Canada, the U.K., and the U.S. (Lua, 2017; Tsai et al., 2019). Differences in CPC between countries might be related to varying competition levels between advertisers in those countries since Facebook Ads work like an auction, where advertisers must bid against each other to secure an advertising placement (Ali et al., 2019; Chieruzzi, n.d.).

### 4.2 Study strengths and limitations

This research represents a secondary analysis of the Untire App Study, entailing both strengths and limitations. Strengths are its large scale and international focus on the effectiveness of an innovative mHealth intervention, which was precisely why we a priori chose to recruit participants online via Facebook Ads. While the primary study provides insights into the app's effectiveness, this secondary analysis reveals valuable information about the online recruitment strategy. Specifically, we learned about the reach and patient characteristics of participants interested in (our) mHealth research and the costs of recruiting participants online to achieve a fully powered study.

We believe that comparing different forms of online recruitment (e.g., via other social media channels) and traditional recruitment (e.g., referrals via general practitioner or flyering) would have yielded valuable insights. However, we did not include a direct comparison in our RCT design for this secondary analysis. Yet, we can assume that the costs of hiring research staff across four English-speaking countries across the globe skilled to distribute flyers and screen potential participants are likely more expensive and time-intensive than the time and personnel needed for recruitment via Facebook.

As the use of Facebook declines among youth, future studies should concern the feasibility and effectiveness of recruiting study participants exploring and comparing Facebook social media recruitment with other modalities (e.g., Instagram, Snapchat). A recent cross-sectional study comparing those modalities found that CPC rates ranged between 0.25 and 0.37 USD across Facebook, Instagram, and Snapchat (Ford et al., 2019). This study further indicated that Instagram and Snapchat, in addition to Facebook, may provide a modern, convenient, and cost-effective method to reach younger participants for mHealth research. Further, future studies should explore the influence of saturation (i.e., how often ads are posted), design (i.e., the content of the Ad), as well individual factors (i.e., who is likely to respond to the Ad) on converting Facebook Ad engagement into study participation (Wozney et al., 2019).

Concerning the study findings' representativeness, the study sample reached is limited to Facebook-users. Further, the finding that women are easier to target than men appears to be a commonality in (m)health and oncology research. Strategies to improve male participant engagement might involve targeting social media networks of men (i.e., partners, family, and friends) to motivate study participation. Interestingly, recent research demonstrated that using male-specific advertisements can result in a significantly higher proportion of men completing the survey as compared to gender-neutral Ads (male-specific Ads: 38% vs. gender-neutral Ads: 25%; *P* < .001) (Lee et al., 2020). Our finding on participants' education levels (high = 42%, moderate = 7%, low = 21%) is in line with current research in the field (Lee et al., 2020), indicating that more people with higher education levels tend to engage in (m)health research studies in general, possibly due to greater levels of health literacy as well as digital mobile phone literacy. The distribution of participants' cancer diagnoses (i.e., 79% breast cancer) is in line with other international Facebook recruitment research in the field of oncology (Tsai et al., 2019).

Apart from recruitment via advertisements on Facebook using Facebook Ads Manager, we also aimed to disseminate our study-link on online patient support platforms and websites. This secondary pathway was unsuccessful since many providers demanded high fees to publish our advertisements, perhaps since the Untire app was initially available for purchase during the study start and only free for study participants. Therefore, we neglected this pathway and decided to solely spend our financial resources on Facebook recruitment via Facebook Ads Manager.

### 4.3 Conclusions

Online recruitment via Facebook Ads Manager can be a suitable strategy in broadening the reach for large-scale clinical RCTs specifically for novel mHealth interventions, reaching patients directly online at the source (smartphone) where the mHealth intervention is provided. One should not underestimate the costs, but we believe this online recruitment strategy is useful to reach participants who represent the end-users, providing high ecological validity of the research findings.

The following is the supplementary data related to this article.

Supplement 1 Samples of Ad creatives used over time with varying pictures, texts, and formats. Supplement 1

## Declaration of competing interest

None declared. The University Medical Center Groningen received funding from Tired of Cancer BV., the Untire app developer, to study its effectiveness independently. Independence is declared in a research agreement.
