# Supplementary material for: Recruiting participants for an international mHealth study via Facebook Ads: Experiences from the Untire App RCT
Source: Internet Interv. 2021 Jan 7;23:100362. doi: 10.1016/j.invent.2021.100362 (PMC7811041; doi:10.1016/j.invent.2021.100362)
Supplement: Supplement 1 — Samples of Ad creatives used over time with varying pictures, texts, and formats. [file mmc1.docx]

Appendix
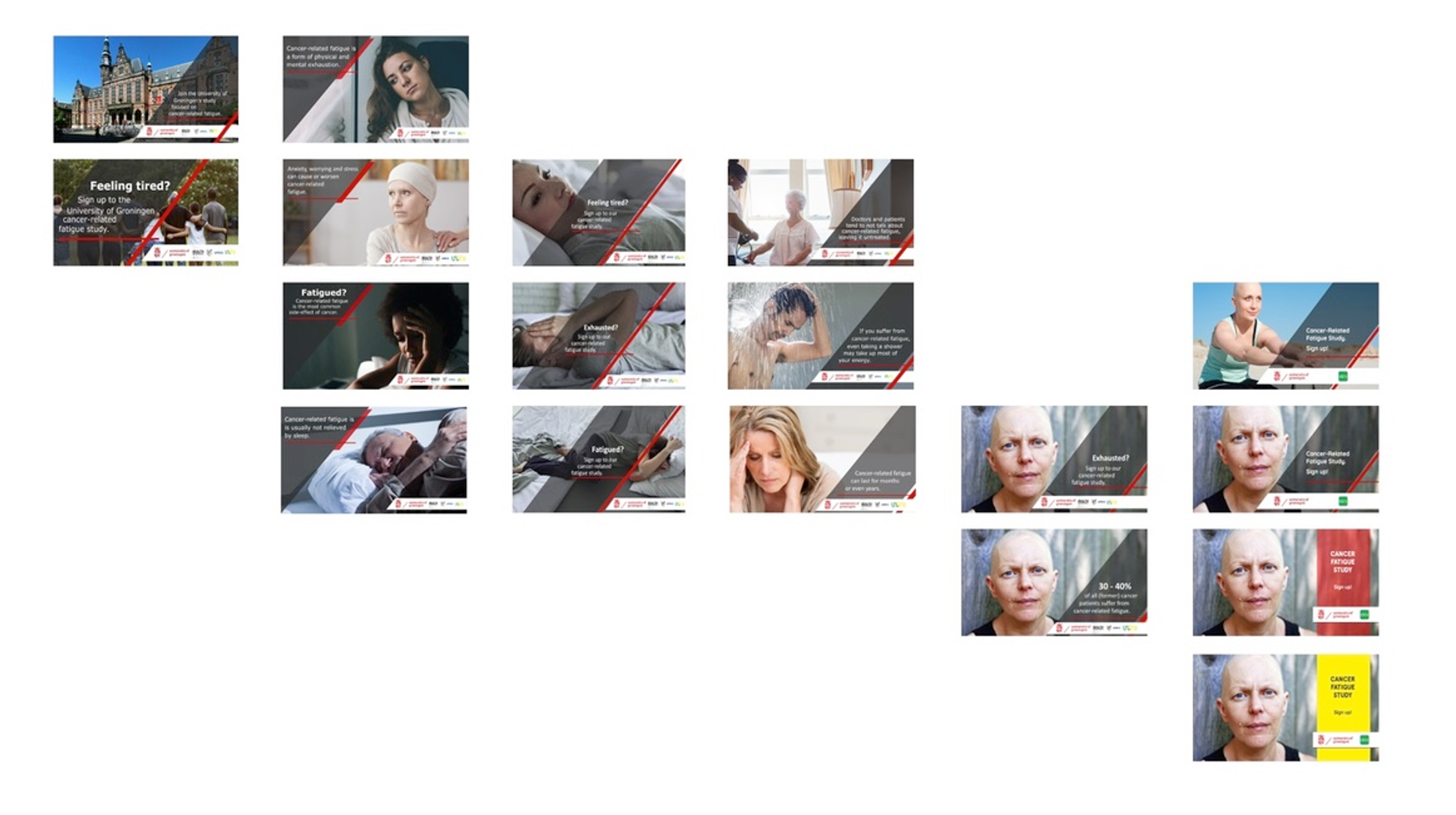


*Supplement 1.* Samples of Ad creatives used over time with varying pictures, texts, and formats.
